# Broad Protection of Pigs against Heterologous PRRSV Strains by a GP5-Mosaic DNA Vaccine Prime/GP5-Mosaic rVaccinia (VACV) Vaccine Boost

**DOI:** 10.3390/vaccines8010106

**Published:** 2020-02-28

**Authors:** Junru Cui, Caitlin M. O’Connell, Connor Hagen, Kim Sawicki, Joan A. Smyth, Paulo H. Verardi, Herbert J. Van Kruiningen, Antonio E. Garmendia

**Affiliations:** Department of Pathobiology and Veterinary Science, College of Agriculture, Health and Natural Resources, University of Connecticut, Storrs, CT 06269, USA; junru.cui@uconn.edu (J.C.); connor.hagen@uconn.edu (C.H.); kimitt@gmail.com (K.S.); joan.smyth@uconn.edu (J.A.S.); paulo.verardi@uconn.edu (P.H.V.); herbert.vankruiningen@uconn.edu (H.J.V.K.)

**Keywords:** PRRSV Mosaic T-cell DNA vaccine VACV, PRRS, cross protection, heterologous virus challenge

## Abstract

Background: Porcine reproductive and respiratory syndrome (PRRS) viruses are a major cause of disease and economic loss in pigs worldwide. High genetic diversity among PRRSV strains is problematic for successful disease control by vaccination. Mosaic DNA and vaccinia (VACV) vaccines were developed in order to improve protection against heterologous PRRSV strains. Methods: Piglets were primed and boosted with GP5-Mosaic DNA vaccine and recombinant GP5-Mosaic VACV (rGP5-Mosaic VACV), respectively. Pigs vaccinated with rGP5-WT (VR2332) DNA and rGP5-WT VACV, or empty vector DNA and empty VACV respectively, served as controls. Virus challenge was given to separate groups of vaccinated pigs with VR2332 or MN184C. Necropsies were performed 14 days after challenge. Results: Vaccination with the GP5-Mosaic-based vaccines resulted in cellular reactivity and higher levels of neutralizing antibodies to both VR2332 and MN184C PRRSV strains. In contrast, vaccination of animals with the GP5-WT vaccines induced responses only to VR2332. Furthermore, vaccination with the GP5-Mosaic based vaccines resulted in protection against challenge with two heterologous virus strains, as demonstrated by the significantly lower viral loads in serum, tissues, porcine alveolar macrophages (PAMs), and bronchoalveolar lavage (BAL) fluids, and less severe lung lesions after challenge with either MN184C or VR2332, which have only 85% identity. In contrast, significant protection by the GP5-WT based vaccines was only achieved against the VR2332 strain. Conclusions: GP5-Mosaic vaccines, using a DNA-prime/VACV boost regimen, conferred protection in pigs against heterologous viruses.

## 1. Introduction

Porcine reproductive and respiratory syndrome (PRRS) is a major disease in pigs that causes significant economic losses to industry across the world. In the United States alone, PRRS causes over $600 million in losses a year [1]. PRRS causes reproductive failure in pregnant sows and respiratory disease in young pigs. The causal virus, PRRSV, is a positive-sense, single-stranded RNA virus with a lipid envelope, and belongs to the Family *Arteriviridae,* Order *Nidovirales* [2,3,4]. The genome is approximately 15 Kb long and encodes at least 22 different viral proteins including fourteen non-structural and eight structural proteins [5]. PRRSV induces both humoral and cellular immune responses in pigs and some of the viral proteins inducing such responses have been identified; however, protection against reinfection is incomplete [6,7,8,9]. Killed virus (KV) vaccines and modified live virus (MLV) vaccines for PRRS have been licensed for more than two decades. These vaccines generally reduce the severity of clinical signs and the transmission of virus; however, they do not induce sterilizing immunity. In general, the efficacy of MLV vaccines is superior to that of KV vaccines [10,11,12,13]. Importantly, however, highly variable and generally sub-optimal levels of protection against heterologous PRRSV strains have been reported [10,14,15,16,17,18]. The potential for reversion to the virulence of MLV vaccines and subsequent transmission to susceptible pigs is a concern [19,20,21].

The major challenge for the development of efficacious, broadly protective PRRS vaccines is the extraordinary genetic variation among disease producing PRRSV strains. Currently, two major PRRSV genotypes are recognized, Genotype 1 (European) and Genotype 2 (North American), which exhibit nearly 40% sequence dissimilarity [22,23]. Genotype 1 and Genotype 2 PRRSVs can be further divided into four subgroups and nine different lineages, respectively, based on phylogenetic analysis of ORF5 [24]. The co-existence of multiple variants within one farm, or even within individual pigs, possibly indicates the occurrence of quasispecies variation of PRRSV [25]. Thus, the development of vaccines that can induce protection against different subgroups and lineages is highly desirable. Multi-subunit vaccines [26], consensus vaccines [27], molecular breeding of different viral structural proteins [28], Mosaic T-cell vaccines [29,30], a polyvalent vaccine containing five different live-attenuated PRRSV strains [31] and DNA prime-MLV boost [32] are just some of the vaccines that are or have been under evaluation recently.

Studies on potential vaccines against human immune deficiency virus type 1 (HIV-1), have shown that Mosaic sequences designed from naturally occurring HIV sequences could effectively address genetic diversity when used as vaccines [33,34]. Mosaic vaccines have been shown to elicit broader immune responses successfully and to confer cross protection in non-human primates and these Mosaic HIV vaccines have been trialed in humans [34,35,36,37,38]. We previously reported that a PRRSV Mosaic vaccine based on 748 GP5 sequences of the Genotype 2 PRRSV strain was immunogenic [29] and induced reactivity to four divergent Genotype-2 PRRSV strains that had at least 10% or more difference in their GP5 sequence—as shown by the expression of interferon gamma (IFN-γ) by peripheral blood mononuclear cells—and, further, that vaccinated pigs were protected against challenge with VR2332 [30]. The present study demonstrates further that vaccination of pigs with the GP5-Mosaic by using a DNA prime and rVaccinia boost approach protects them against strains that are more than 10% divergent in their sequences (VR2332 and MN184C).

## 2. Materials and Methods

### 2.1. Viruses and Cells

The viruses used in the study included the VR2332 NA reference strain (ATCC VR-2332) and the MN184C strain provided kindly by Drs. Kelly Lager and Kay Faaberg at U.S. Department of Agriculture-Agriculture Research Service (USDA-ARS). The viruses were propagated in MARC-145 cells [39] grown in Dulbecco’s modified Eagle’s medium (DMEM) containing 2 mM L-glutamine, 100 U penicillin/mL, 100 µg streptomycin/mL and 10% fetal bovine serum (FBS). The Reed–Muench formula was used to calculate virus titers [40]. Viruses were purified over continuous cesium chloride gradients, quantified using a NanoDrop 1000 spectrophotometer (Thermo Scientific, Waltham, MA, USA) and stored at −80 °C for later use. Titrated viruses were used for ex vivo recall immune response assay and challenge experiments.

### 2.2. Transfer Vector Construction

A combination of gene synthesis (DNA 2.0, Menlo Park, CA, USA) and standard sub-cloning was utilized to generate three transfer vectors. Each transfer vector contained a cassette with the tetR gene (based on GenBank: X00694), and either a natural or Mosaic version of Genotype 2 PRRSV ORF 5 [29,30], followed by the EMCV IRES (based on GenBank: NC_001479.1) and the EGFP gene (based on plasmid pEGFP-1, GenBank: U55761). The tetR gene and the ORF 5–IRES-EGFP genetic segment were placed under back-to-back synthetic VACV early/late promoters [41]. The tetO2 operator sequence was inserted after the putative VACV D6R promoter, as described in [42]. The cassettes were flanked by 600 bp of the VACV D5R gene to the left and 600 bp of the VACV D6R gene to the right (based on GenBank: NC_006998.1). Restriction endonuclease analysis was used to confirm plasmid identity.

### 2.3. Generation of Recombinant VACVs and Preparation of High-Titer Stocks

Standard homologous recombination was used to generate Tetracycline-inducible recombinant VACVs after the transfection of transfer vectors with FuGene HD Transfection Reagent (Promega, Madison, WI, USA) into BS-C-1 cells, infected 1 h before with an IPTG-inducible VACV strain (based on the WR clone 9.2.4.8.) in the presence of 0.1 mM IPTG and 1 µg/mL doxycycline. Recombinant EGFP-positive tetracycline-inducible VACVs were plaque purified in the absence of Isopropyl β- d-1-thiogalactopyranoside (IPTG) and in the presence of 1 µg/mL doxycycline. The elimination of the parental virus was confirmed by PCR analysis of viral DNA that was purified using a Nucleospin^®^ Blood kit (Macherey-Nagel, Düren, Germany). High-titer stocks were generated by infecting HeLa S3 cells with recombinant virus at a multiplicity of infection (MOI)of 0.1 in the presence of 1 µg/mL doxycycline. Infected cells were harvested and homogenized 4 days post-infection. Homogenates were clarified by centrifugation at 750× *g* for 10 min, purified over a sucrose cushion [43], and resuspended in 1X PBS pH 7.3 (with no doxycycline).

### 2.4. Protein Sequence Alignment and Phylogenetic Analysis

MEGA 7 (Molecular Evolutionary Genetics Analysis) (www.megasoftware.net) (NIH, Bethesda, MD, USA) was utilized to evaluate protein sequence alignments between GP5 sequences of VR2332, MN184C, Mosaic 1 and Mosaic 2 and to create a phylogenetic tree.

### 2.5. Vaccination and Collection of Samples

Cross-bred three- to four-week-old male and female piglets, which were free of PRRSV and porcine circovirus-2, were used in this study. The experimental design is summarized in Table 1. GP5-Mosaic, GP5-WT and vector-control vaccines were administered by both intradermal and intramuscular injection. Briefly, 1 mL vaccine containing 500 μg DNA and 100 μg Quil-A^®^ as adjuvant was injected intradermally (0.1 mL) on the back of ear and intramuscularly (0.9 mL) in the neck region at day 0 and again on day 14. The animals were further boosted at day 28 with a 1 mL suspension containing 10^8^ PFU VACV expressing GP5-Mosaic, GP5-WT or VACV and 100 μg Quil-A^®^ as adjuvant. Pigs were challenged at day 35 with VR2332 and at day 37 with MN184C to their respective groups. A total of 10^6^ TCID_50_ of each virus was administered by intranasal and intramuscular challenge to each animal. Blood was collected at days 0, 7, 14, 21, 28, the challenge day, and 4, 7, 10, and 14 days after challenge.

The pigs were euthanized at 14 days post-challenge. The lungs were evaluated macroscopically, weighed, and bronchoalveolar lavage (BAL) was then performed. Tissue samples were collected from each lung lobe, tracheobronchial lymph nodes (TBLN), spleen, inguinal lymph nodes (ILN) and tonsils: portions of each were either fixed in 10% neutral buffered formalin or kept frozen. Fixed tissues were routinely processed to paraffin, and 4–5 µm sections were stained with hematoxylin and eosin and evaluated histologically. Lung lesion scores were calculated as reported before [44]. All animal work was performed under a protocol approved by the University of Connecticut Institutional Animal Care and Use Committee, the animal protocol number under which the study was conducted is IACUC A17-003.

### 2.6. Measurement of IFN-γ Immune Response to PRRSV

Peripheral blood mononuclear cells harvested from blood collected at days 0 and on the challenge day, were seeded in 24-well flat-bottom plates (5 × 10^5^ cells/well). They were then stimulated, in duplicate, by adding 200 TCID_50_ VR2332 or MN184C/well, or mock treated for 48 h at 37 °C in a 5% CO_2_ atmosphere. The cells were then collected and total RNA was extracted for quantitative real-time PCR analysis.

### 2.7. Serum Neutralization Test

Virus neutralization testing was carried out as previously reported [30]. Briefly, each serum was mixed with equal volumes of DMEM containing 100 TCID_50_ of VR2332 or MN184C and the mixtures were incubated at 37 °C for 1 h. The serum–virus mixtures were then added to 96-well plates containing 80–90% confluent MARC-145 cells and incubated for 48 h at 37 °C in a 5% CO_2_ atmosphere (final serum dilution 1:4). The VR2332 or MN184C virus, negative serum, and uninfected cells served as the virus control, negative serum control and cell control, respectively. The neutralizing capacity of serum was quantified by measuring the viral copy numbers by RT-qPCR in supernatants 48 h after the addition of pre-incubated serum–virus mixtures to the cells.

### 2.8. Quantitative Real-Time PCR

TRIzol LS Reagent or TRIzol Reagent (Invitrogen, Grand Island, NY, USA) was used to extract total RNA from serum/ culture supernatants or tissues, respectively. The extracted RNA was quantified in a NanoDrop 1000 spectrophotometer (Thermo Scientific, Waltham, MA, USA). The cDNA was synthesized from each sample RNA using a 20 µL reaction mixture and random primers (Invitrogen, Grand Island, NY, USA) and the following reaction conditions: 26 °C for 10 min, 42 °C for 45 min and 75 °C for 10 min. The cDNA was then amplified by SYBR Green real-time PCR. To this effect, SYBR qPCR Master Mix (Bimake, Huston, TX, USA), the cDNA template and 5′-ATG ATG RCC TGG CAT TCT-3′and 5′-ACA CGG TCG CCC TAA TTG-3′ were utilized as the forward and reverse primers for ORF7, respectively. Real time PCR was then performed as follows: 2 min at 95 °C, followed by 40 cycles at 95 °C for 15 s and 61 °C for 1 min using Bio-Rad CFX96 Touch System (Bio-Rad, Hercules, CA, USA). For use in quantification, a standard curve was generated using serial dilutions of viral RNA which contained 10^2^–10^7^ copies/μL. Both positive and negative reference samples were run concurrently with the test samples. Viral loads were determined by plotting the Ct values against the standard curve. Melting curves were analyzed to verify the specificity of the PCR.

To test for IFN-γ expression, total RNA was extracted by TRIzol Reagent from virus-stimulated or mock-treated PBMCs; this was then used as template for cDNA synthesis and this, in turn, was used for real-time PCR following the protocol described above. Glyceraldehyde 3-phosphate dehydrogenase (GAPDH) (forward primer: 5′-CGT CCC TGA GAC ACG ATG GT-3′and reverse primer: 5′-CCC GAT GCG GCC AAA T-3′) was used as internal control to calculate the fold changes in the expression of IFN-γ (forward primer: 5′-TGG TAG CTC TGG GAA ACT GAA TG-3′and reverse primer: 5′-GGC TTT GCG CTG GAT CTG-3′) by the delta–delta method [44].

### 2.9. Lung Lesion Scoring

The scoring of lung lesions was done systematically, as previously described [45], by a board-certified veterinary pathologist, blinded to the treatment groups. Nine sections, representing all six lung lobes, were scored, with one section from each division of the left cranial lung lobe and two sections from each diaphragmatic lobe.

### 2.10. Statistical Analysis

Two-way ANOVA or Student’s t-test was used to evaluate the differences in measurements between the samples within or between groups. The data were analyzed using GraphPad Prism (version 7.0) (GraphPad Software, San Diego, CA, USA).

## 3. Results

### 3.1. Sequence Alignment and Analysis of GP5

**Figure 1:**
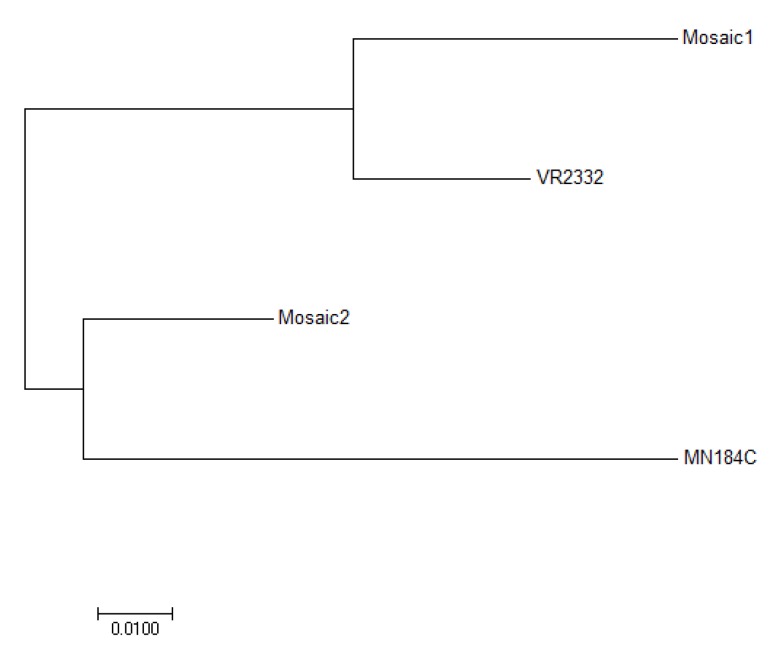
Phylogenetic analysis of the GP5 Amino acid sequence of the two Mosaic sequences, MN184C and VR2332. The analysis was done via the neighbor-joining method using MEGA7.0.

The GP5 amino acid (aa) sequences in Mosaic 1, Mosaic 2, MN184C and VR2332 (the prototype of Genotype 2 PRRSV) were all the same size, with no deletions or insertions. Sequence alignments showed aa identity from 85% to 93%. To further investigate the antigenic relationship of these strains, a phylogenetic tree was constructed using the neighbor-joining method b. The four sequences clustered into two subgroups (Figure 1), with the two most distant being Mosaic 1 and MN184C. Mosaic 1 and Mosaic 2 were, relatively, more closely related to VR2332 and MN184C, respectively.

### 3.2. GP5-Mosaic Vaccines Induced Both Humoral and Cellular Response

**Figure 2:**
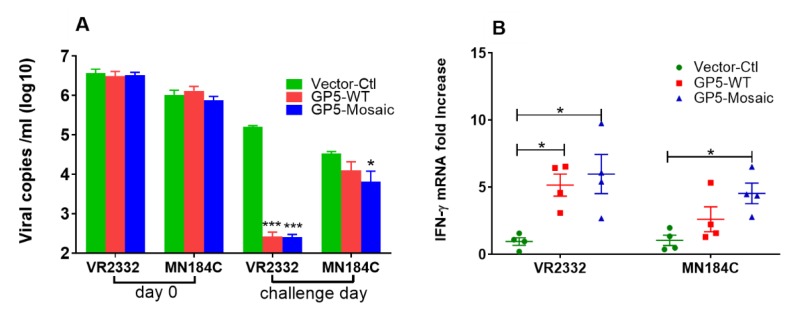
Vaccination-induced humoral and cellular responses. (**A**) The virus neutralization was expressed as viral copy numbers (log10 scale), as measured by RT-qPCR in cell supernatants after the infection of MARC-145 cells with pre-incubated serum–virus mixtures. (**B**) The expression of IFN-γ mRNA as fold changes, by either VR2332 or MN184C-stimulated PBMCs collected on the challenge day. Each dot represents the value of one animal. The variation is expressed as standard error of the means. There were three independent replications. Significant differences were calculated by a two-way ANOVA or Student’s *t* test (* *p* < 0.05, *** *p* < 0.0001).

GP5-Mosaic-vaccinated pigs had significantly higher levels of neutralizing antibodies in their serum compared to the vector-control animals, both to VR2332 (*p* < 0.001) and MN184C (*p* < 0.05) The virus neutralizing capability of serum from GP5-WT-vaccinated animals was greater against VR2332 (*p* < 0.01) and against MN184C (*p* > 0.05) when compared to serum from vector control animals (Figure 2A); vaccination with the GP5-Mosaic vaccine resulted in broader recall cellular responses than those induced with the GP5WT vaccine. Thus, a significantly higher (*p* < 0.05) relative fold-change in IFN-γ mRNA expression was detected upon stimulation of PBMCs derived from GP5-Mosaic-vaccinated pigs, with either VR2332 or MN184C strains, when compared to those in equally stimulated PMBCs from vector control pigs (Figure 2B). In contrast, the expression of IFN-γ mRNA in GP5-WT-vaccinated pigs was significantly higher only if their PBMCs were stimulated with VR2332, when compared to those in the equally stimulated PBMCs of the control pigs at the same time points (Figure 2B). No changes were detected with the PBMCs of vector-control pigs with any type of stimulation.

### 3.3. GP5-Mosaic Vaccination Induced Cross-Protection in Pigs

**Figure 3:**
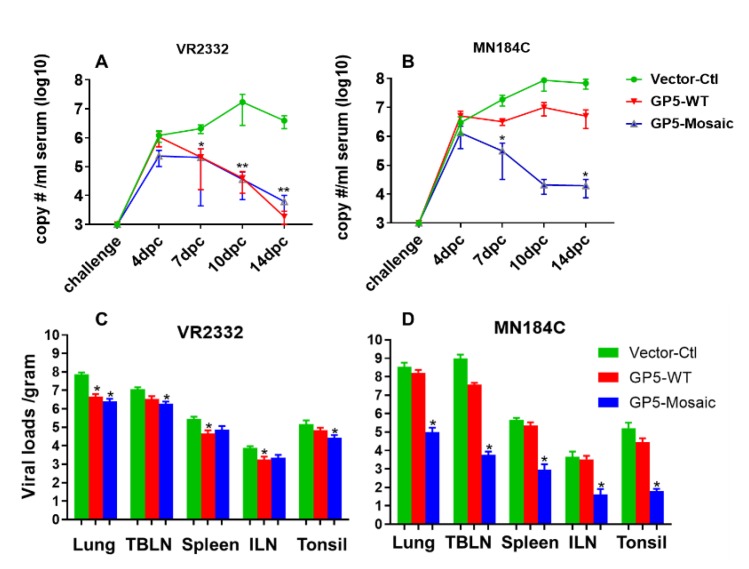
Virus clearance in sera and tissues. (**A**) Viral copy numbers in serum from 0 to 14 DPC upon VR2332 challenge. (**B**) The viral copy numbers in serum from 0 to 14 DPC upon MN184C challenge. Each dot represents the mean value of each group. The variation bars are expressed as the standard error of the mean. Three separate experiments were performed for each. A two-way ANOVA or Student’s *t* test (* *p* < 0.05, ** *p* < 0.01, was used to calculate significant differences; days post-challenge (DPC). (**C**) The viral copy numbers in tissues at necropsy upon VR2332 challenge. (**D**) The viral copy numbers in tissues at necropsy upon MN184C challenge. Each bar represents the mean value of each group. The bars are the standard error of the mean. Three separate experiments were performed for each. Significant differences were calculated by Student’s *t* test (* *p* < 0.05).

Viral loads in serum of both GP5-WT and GP5-Mosaic-vaccinated groups were significantly lower than those in the vector-control group at 7, 10 and 14 days after challenge with VR2332 (* *p* < 0.05, ** *p* < 0.01) (Figure 3A). Therefore, the capacity of reducing VR2332 virus loads in serum was relatively similar between the GP5-Mosaic and the GP5-WT vaccines. Furthermore, there was a steady decrease in viral loads from 4 days to 14 days after challenge in both GP5-WT and GP5-Mosaic vaccinated groups, while viral loads increased during the course of challenge and reached their peak at 10 days post-challenge (DPC), then declining slightly at 14 DPC in the vector-control group (Figure 3A). In contrast, the viral loads in serum of animals receiving the GP5-Mosaic-vaccine were significantly lower than those of animals receiving the GP5-WT vaccine or those of vector-control animals at 7 and 14 DPC with MN184C (* *p* < 0.05) (Figure 3B), which was different compared to VR2332-challenged groups. Viral load levels in GP5-WT vaccinated animals, increased during the infection with MN184C and reached their peak at 10DPC, which is comparable to the vector-control group. The viral loads in GP5-Mosaic vaccinated animals decreased steadily from 4 to 10 DPC and remained unchanged until 14 DPC (Figure 3B). Furthermore, the viral loads in lung, TBLN, spleen, ILN and tonsils were significantly lower in GP5-Mosaic-vaccinated animals than those in the vector-control group (* *p* < 0.05) after challenge with MN184C, while those of the GP5-WT group were similar or slightly lower compared to the vector-control group (Figure 3C). Viral loads in the lungs, TBLN, and tonsil were significantly lower in GP5-Mosaic-vaccinated animals than those in the vector-control group (* *p* < 0.05) after challenge with VR2332, while those of the GP5-WT group were significantly lower in the lungs, spleen and ILN compared to the vector-control group (* *p* < 0.05). There was no significant difference in VR2332 virus loads between the GP5-Mosaic and GP5-WT groups (Figure 3D).

**Figure 4:**
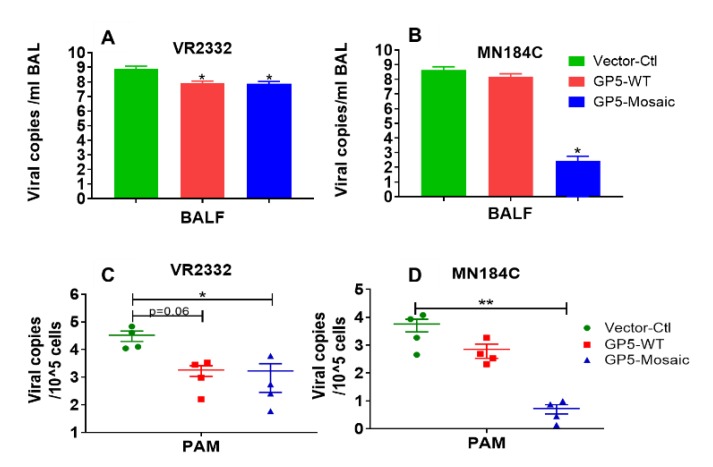
Virus clearance in bronchioalveolar lavage fluids (BAL) and PAMs. (**A**) The viral copy numbers in BAL fluids at necropsy upon challenge with VR2332. (**B**) The viral copy numbers in BAL fluids at necropsy upon challenge with MN184C. Each bar represents the mean value of each group. Bars represent the standard error of the mean. Three independent experiments were performed for each. Significant differences were calculated by Student’s *t* test (* *p* < 0.05). (**C**) The viral copy numbers in PAMs at necropsy upon challenge with VR2332. (**D**) The viral copy numbers in PAMs at necropsy upon challenge with MN184C. Each dot represents the mean value of each animal. The bars represent the standard error of the mean. Three independent experiments were performed for each. Student’s *t* test was used to calculate significant differences (* *p* < 0.05, ** *p* < 0.01).

Tests in PAMs and BAL fluids showed a similar pattern in which the viral loads in both the GP5-Mosaic and GP5-WT groups were lower than those in the vector-control group after challenge with VR2332 (Figure 4A,C), meanwhile only the viral loads of GP5-Mosaic-vaccinated animals were significantly lower than those in the vector-control animals after challenge with MN184C (* *p* < 0.05 and ** *p* < 0.01, respectively), furthermore there was no apparent difference in MN184C viral loads between GP5-WT and vector-control animals (Figure 4B,D).

### 3.4. Lower Lung Lesion Scores Detected in GP5-Mosaic-Vaccinated Animals

**Figure 5:**
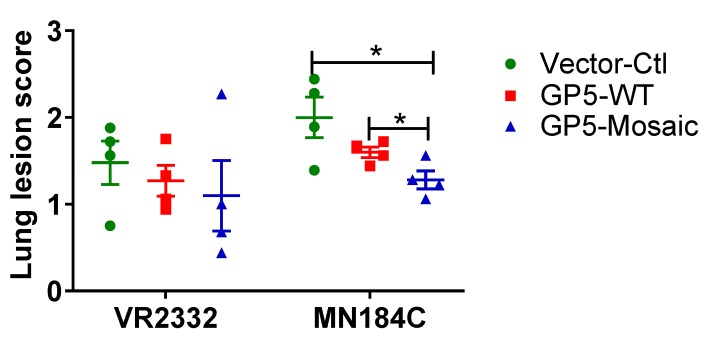
Lung lesion scores. The lung lesion scores of GP5-Mosaic-vaccinated animals were significantly lower (*p* < 0.05) than those of GP%-WT or vector-control animals after challenge with MN184C. Each figure represents the mean value of each individual. The bars represent the standard error of the mean. Three independent experiments were performed for each. Student’s *t* test was utilized to calculate significant differences (days post-challenge (DPC)).

The lung lesion scores after challenge with MN184C were significantly lower in GP5-Mosaic-vaccinated animals than those in both GP5-WT and vector-control animals when evaluated using nine sections of lung (Figure 5 right panel, *p* < 0.05). In contrast, after VR2332 challenge, lung lesion scores were lower on average in both the GP5-Mosaic and GP5-WT groups than those in vector-control animals. There were no significant differences between the lung lesion scores of GP5-WT and GP5-Mosaic-vaccinated groups after challenge with VR2332 (Figure 5).

## 4. Discussion

There is extraordinary diversity among PRRSV strains. The diversity among PRRSV strains is as high as, or may even surpass that of, HIV. Recently, several vaccine candidates, including a synthetic consensus PRRSV strain [27], a chimeric PRRSV strain containing multiple ORFs DNA shuffled [46,47,48] and an intranasal live virus vaccine with adjuvant [49] induced various levels of heterologous protection in pigs. We have developed GP5-Mosaic vaccines that incorporate sequences derived from naturally circulating viruses. The data obtained from our GP5-Mosaic vaccinated pigs supports the Mosaic vaccine approach as an effective strategy to address PRRSV diversity. It has also been demonstrated that Mosaic T-cell vaccines have great potential for other viruses with extraordinary diversity, such as HIV [33,34,35,36,37,38]. In an earlier study, we demonstrated the immunogenicity of GP5-Mosaic vaccines in swine and their ability to induce cross-reactivity, as shown by their broad recall responses ex-vivo, and conferred the partial protection they provide in pigs [29,30]. The ability of the GP5-Mosaic vaccines to induce cross-protection in pigs against heterologous PRRSV strains was confirmed in the present study.

A sequence analysis of two GP5-Mosaic vaccines and the two Genotype 2 PRRSV strains utilized in the study, VR2332 and MN184C, that belong to lineage 5 and to lineage 1, respectively, revealed a 15% difference in the GP5 aa sequences. The aa sequences were used to generate a phylogenetic tree using the neighbor-joining method [24]. The GP5-Mosaic 1 was closer to VR2332 (93% aa identity), while GP5-Mosaic 2 was closer to MN184C (90% aa identity), which suggested that the two GP5-Mosaic sequences broaden the coverage to strains belonging to different lineages. Based on this analysis, a broader protection could be expected with GP5-Mosaic vaccines. The data of the present study supports clearly the capacity of the GP5-Mosaic vaccine to cross-protect pigs against VR2332 and MN184C that share only 85% aa identity.

VACV provides a very good platform for the development and testing of vaccines, including animal vaccines, human vaccines and cancer immunotherapy [50]. In our study, a GP5-Mosaic DNA vaccine prime/GP5-Mosaic rVaccinia (VACV) boost regime was used to immunize pigs. This type of prime-boost regime has previously been demonstrated to increase vaccination efficacy substantially [51,52]. In addition, Quil-A^®^, used as adjuvant for the priming of the GP5-Mosaic DNA vaccine, was previously shown to further increase the immune responses [53,54,55]. Therefore, the prime-boost vaccination regime used here for testing GP5-Mosaic vaccine efficacy, in terms of broad protection, proved appropriate for testing the hypothesis.

PBMCs collected on the challenge day from GP5-Mosaic-vaccinated pigs responded to a broader range of virus strains, as measured by IFN-γ mRNA expression, than those from GP5-WT vaccinates. Thus, PBMC from GP5-Mosaic-vaccinated pigs expressed higher levels of IFN-γ mRNA in response to stimulation with VR2332 or MN184C strains, respectively, compared to those of the vector-control pigs. In contrast, PBMC from GP5-WT vaccinated pigs expressed higher levels of IFN-γ mRNA only when stimulated with VR2332. These data were consistent with those of our previous study where the GP5-Mosaic vaccine induced broad recall cellular responses, as measured by significantly higher levels of IFN-γ mRNA expression in response to the four diverse Genotype 2 PRRSV strains tested, including VR2332, NADC9, NADC30 and SDSU73 [30]. The significant induction of IFN-γ is an important asset of a PRRSV vaccine, as IFN-γ reportedly plays a critical role in the control of and protection from PRRSV infection [56]. In addition, both the GP5-Mosaic and the GP5-WT vaccines induced relatively high levels of neutralizing antibodies in VR2332-challenged pigs. These data indicate that the GP5-Mosaic vaccine preserves virus-neutralizing epitopes that appear to contribute to the overall efficacy of this vaccine.

In terms of vaccine-induced protection, both GP5-Mosaic and GP5-WT-vaccinated pigs had significantly lower serum viral loads at 7, 10 and 14 DPC than the vector-control group after the VR2332 challenge. In contrast, only the GP5-Mosaic-vaccinated group showed significantly lower viral loads in sera at 7 and 14 DPC than the vector-control group after the MN184C challenge. These data demonstrate that the GP5-Mosaic vaccine is capable of cross-protecting pigs against two divergent PRRSV strains (15% difference) while the GP5-WT vaccine only provided protection in pigs against the homologous strain. Furthermore, a similar pattern was found with respect to viral loads in tissues, where the GP5-Mosaic-vaccinated pigs had significantly lower viral loads in their lungs, TBLN, spleen, ILN and tonsils compared to the vector-control group after challenge with MN184C, while the GP5-WT group only showed slightly lower (numerically, but not significant) viral loads. In the VR2332 challenge experiment, both the GP5-Mosaic and GP5-WT vaccinates had significantly lower viral loads in tissues generally—the viral loads in the GP5-Mosaic vaccinates were significantly lower in their lungs, TBLN and tonsils, while viral loads in the GP5-WT group were significantly lower in their lungs, ILN and spleen, compared to the vector-control group. Similar protective effects were also demonstrated in the GP5-Mosaic vaccine vaccinates, where lower viral loads were detected in the BAL fluids or PAMs after MN184C challenge, further confirming the cross-protective capability of the GP5-Mosaic vaccine. Indeed, this is finding may be particularly relevant since PAMs are the primary target of PRRSV infection. Lung lesion scoring is an essential and crucial method to evaluate vaccine efficacy, as infection by PRRSV tends to increase susceptibility to coinfection with other virus or bacterial pathogens [57,58,59] which may result in more severe lesions and mortality. Pigs vaccinated with the GP5-Mosaic vaccine had significantly lower lung lesion scores than both the GP5-WT and vector-control groups after challenge with MN184C, while both the GP5-Mosaic and GP5-WT groups had lower lung lesion scores compared to the vector-control group in the VR2332 challenge experiment.

## 5. Conclusions

Taken altogether, our results clearly demonstrate that the GP5-Mosaic vaccine provided cross-protection against two heterologous PRRSV strains, VR2332, the prototype Genotype 2 strain, and MN184C, a highly virulent strain, while the GP5-WT vaccine provided only homologous protection against VR2332. In summary, our results demonstrate that our GP5-Mosaic vaccine is able to induce broader protection against PRRS and warrants further research for field applications.

## Figures and Tables

**Table 1:** Experimental design.

Group	Vaccination ^a^	Challenge ^b^
A (*n* = 8)	DNA/Vector-control/VACV	VR2332 (*n* = 4)
		MN184C (*n* = 4)
B (*n* = 8)	DNA GP5-WT/VACV GP5 WT	VR2332 (*n* = 4)
		MN184C (*n* = 4)
C (*n* = 8)	DNA GP5-Mosaic/VACV GP5-Mosaic	VR2332 (*n* = 4)
		MN184C (*n* = 4)

^a^ One mL vaccine containing 500 μg DNA and 100 μg Quil-A^®^ as adjuvant was injected intradermally (0.1 mL) on the back of ear and intramuscularly (0.9 mL) in the neck region at day 0 and again at day 14. VACV 10^8^ PFU and 100 μg Quil-A^®^ as adjuvant was administered at day 28. ^b^ Pigs were challenged at day 35 with 10^6^TCID_50_ VR2332 (*n* = 4/group) and day 37 with 10^6^TCID_50_ MN184C (*n* = 4/group) both intranasally and intramuscularly.
